# Low-density lipoprotein receptor-related protein 6 is a novel coreceptor of protease-activated receptor-2 in the dynamics of cancer-associated β-catenin stabilization

**DOI:** 10.18632/oncotarget.16246

**Published:** 2017-03-16

**Authors:** Jeetendra Kumar Nag, Arun Kancharla, Myriam Maoz, Hagit Turm, Daniel Agranovich, Chhedi Lal Gupta, Beatrice Uziely, Rachel Bar-Shavit

**Affiliations:** ^1^ Sharett Institute of Oncology, Hadassah-Hebrew University Medical Center, Jerusalem 91120, Israel; ^2^ Department of Biosciences, Integral University, Lucknow, Uttar Pradesh 226026, India

**Keywords:** protease-activated receptors (PARs), protease, G-protein coupled receptors (GPCRs), cancer, β-catenin stabilization

## Abstract

Protease-activated receptor-2 (PAR_2_) plays a central role in cancer; however, the molecular machinery of PAR_2_-instigated tumors remains to be elucidated. We show that PAR_2_ is a potent inducer of β-catenin stabilization, a core process in cancer biology, leading to its transcriptional activity. Novel association of low-density lipoprotein-related protein 6 (LRP6), a known coreceptor of *Frizzleds* (*Fz*), with PAR_2_ takes place following PAR_2_ activation. The association between PAR_2_ and LRP6 was demonstrated employing co-immunoprecipitation, bioluminescence resonance energy transfer (BRET), and confocal microscopy analysis. The association was further supported by ZDOCK *protein-protein* server. PAR_2_-LRP6 interaction promotes rapid phosphorylation of LRP6, which results in the recruitment of Axin. Confocal microscopy of PAR_2_-driven mammary gland tumors *in vivo*, as well as *in vitro* confirms the association between PAR_2_ and LRP6. Indeed, *sh*RNA silencing of LRP6 potently inhibits PAR_2_-induced β-catenin stabilization, demonstrating its critical role in the induced path. We have previously shown a novel link between protease-activated receptor-1 (PAR_1_) and β-catenin stabilization, both in a transgenic (*tg*) mouse model with overexpression of human PAR_1_ (*hPar1*) in the mammary glands, and in cancer epithelial cell lines. Unlike in PAR_1_-G_α13_ axis, both G_α12_ and G_α13_ are equally involved in PAR_2_-induced β-catenin stabilization. Disheveled (DVL) is translocated to the cell nucleus through the DVL-PDZ domain. Collectively, our data demonstrate a novel PAR_2_-LRP6-Axin interaction as a key axis of PAR_2_-induced β-catenin stabilization in cancer. This newly described axis enhances our understanding of cancer biology, and opens new avenues for future development of anti-cancer therapies.

## INTRODUCTION

Despite the growing appreciation of G protein-coupled receptor (GPCR) signal involvement in cancer pathogenesis, very little is known about the role GPCRs play in tumor etiology. GPCRs are seven transmembrane proteins responsible for transducing signals from a diverse range of ligands that affect numerous physiological processes [1–4]. GPCRs regulate many aspects of tumorigenesis, including proliferation, invasion, and survival at the secondary site, as well as several cancer-associated signaling pathways [5]. Emerging large-scale genomic analyses have recently provided further evidence of frequent GPCR alterations in human tumors, including mutations, copy number, altered expression and promoter methylation [6–8]. Determining the contribution of such alterations to cancer initiation and progression remains a significant challenge, that is critical both for discovery of driver oncogenes and for the development of targeted therapeutics.

*Frizzled* (*Fz*) receptors are a subgroup of GPCRs that play a pivotal role in development, tissue homeostasis, and cancer. Activation of the evolutionarily conserved Wnt/β-catenin signaling pathway, also called the canonical Wnt pathway, involves stabilization of β-catenin through binding of Wnt ligands to *Fz* cell surface receptors and low-density lipoprotein-related protein 5/6 (LRP5/6) coreceptors. In the absence of Wnt, the key effector of this pathway, β-catenin, is continuously degraded by the “degradation complex”. This complex is comprised of Axin, adenomatis polyposis coli (APC), glycogen synthase kinase3β (GSK3β), casein kinase1alpha (CK1α), and the E3 ubiquitin ligase subunit β-TrCP1. Axin provides a scaffolding site for GSK3β to phosphorylate the N-terminus portion of β-catenin (after priming by CK1α), thus generating a phosphorylated form of β-catenin, recognized by the ubiquitin ligase adaptor β-TrCP [9, 10]. Wnt stimulation dismantles the degradation complex, thereby leading to the accumulation of unphosphorylated β-catenin. Once β-catenin is stabilized, it translocates to the cell nucleus. There it alters the activity of members of the lymphoid enhancer factor (Lef)/T-cell factor (Tcf), Lef/Tcf family of HMG-box transcription factors acting as transcriptional switches, recruiting various chromatin modifiers and remodelers to Lef/Tcf target genes inducing expression of an array of genes downstream [10, 11]. A wide range of cancers exhibit hyperactive stabilized β-catenin, either due to oncogenic mutations in its N-terminal phosphorylation site or through mutational inactivation of APC or Axin, its negative regulators [11, 12]. Activated β-catenin can be oncogenic, driving the onset of a wide spectrum of carcinomas [9, 10].

In addition to its pathological role in cell nuclei as a central transcriptional coactivator for Wnt-responsive genes [10, 11], β-catenin is also a membrane-associated protein that constitutes a key component of adherens junctions. Under normal conditions, it has a robust engagement of neighboring adherens junctions and then interacts with the cytoplasmic tail of cadherins [13].

Mammalian protease-activated-receptors PARs, comprise a four-member family subgroup of GPCRs. They are uniquely activated by cleavage of their N-terminal extracellular domain and exposure of internal ligands. PAR_1_ and PAR_2_ play central roles in tumor biology [14–19]. Increasing evidence supports the notion that PAR_1_ and PAR_2_ exist in close proximity and act as one functional unit, forming PAR_1_-PAR_2_ heterodimer [20–23]. We have recently demonstrated that PAR_1_-induced breast tumor development and the corresponding signaling events are markedly inhibited when PAR_2_ expression is knocked-down or when it lacks its C-tail portion [24]. This establishes a dominant role for PAR_2_, which seems essential also for PAR_1_ function, in a manner that is yet unknown. Previously, we have shown a novel link between human protease-activated receptor-1 (*hPar-1*) and β-catenin stabilization using a transgenic mouse model whereby *hPar1* is overexpressed in the mammary glands and several cancer cell-lines [25–27], demonstrating that PAR_1_ induces β-catenin stabilization via *early* formation of the PAR_1_-Gα_13_-DVL-DIX axis. Whereas the role of PAR_1_ in β-catenin stabilization has been addressed, the involvement of PAR_2_ in this process has not been examined.

In the present study, we demonstrate that PAR_2_ activation leads to potent β-catenin stabilization accompanied by increased Lef/Tcf transcription activity. The molecular path that links PAR_2_ with β-catenin stabilization was assessed, identifying LRP6, a known *Fz* partner, as a novel co-receptor of PAR_2_ as shown by co-immunoprecipitation, bioluminescence resonance energy transfer (BRET), and confocal microscopy analysis, and supported by bioinformatics using the ZDOCK *protein-protein* server. Following SLIGKV activation, PAR_2_ forms a complex with LRP6, which then recruits Axin from the “destruction-complex” pool, leading to β-catenin stabilization and nuclear transcriptional activity. We hereby identify the components that link PAR_2_ with β-catenin stabilization and characterize PAR induced DVL nuclear localization. Taken together, our data show a novel PAR_2_-LRP6-Axin axis as key components in PAR_2_-induced β-catenin stabilization in cancer. This first described axis enhances our understanding in cancer biology, and opens new avenues for future development of an anti-cancer medicament platform.

## RESULTS

### PAR_2_ induces β-catenin stabilization and Lef/Tcf transcriptional activity: identification of the minimal PAR_2_ C-tail region

**Figure 1:**
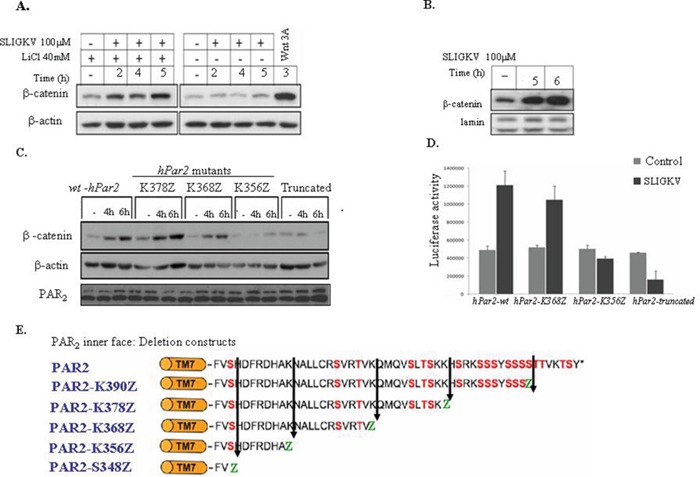
Activation of PAR2 induces β-catenin stabilization and Lef/Tcf transcriptional activity **(A)** PAR_2_ activation induces β-catenin stabilization in RKO cells. RKO cells were pretreated with LiCl (40 mM, 2h) followed by 2, 4, and 5 hours of PAR_2_ SLIGKV activation (100 μM). Whereas low levels of β-catenin were observed prior to PAR_2_ activation, a marked increase in β-catenin levels was seen following activation. RKO cells that were not pretreated with LiCl showed basal levels of β-catenin regardless of PAR_2_ activation. RKO cells pre-treated with Wnt3A conditioned medium for 3 hours were used as a positive control. **(B)** PAR_2_ activation induces nuclear β-catenin in HEK-293T cells. HEK-293T cells were transiently transfected with both *flg*-β-catenin and *hPar2*-*wt* plasmids. After PAR_2_ activation, the nuclear fraction was extracted and immunoblots were analyzed using anti-*flg* (for *flg*-β-catenin) and anti-lamin (used as a nuclearprotein loading control) antibodies. The amount of nuclear β-catenin was markedly increased in response to PAR_2_ activation. **(C)** PAR_2_-induced β-catenin stabilization depends on an intact K356 - K368 region. HEK-293T cells transiently transfected with *hPar2* deletion constructs lacking the K356-K368 (i.e., *hPar2*-*K356Z* or *hPar2-truncated*) displayed a markedly reduced β-catenin stabilization level. In contrast, cells transfected with *hPar2* constructs that possess the above-mentioned region (e.g., *hPar2-K368Z/K378Z* or *wt*) displayed increased β-catenin accumulation. **(D)** An intact K356 - K368 region is essential for PAR_2_-induced Lef/Tcf transcriptional activity. TOPflash luciferase transcription activity was analyzed in RKO cells following PAR_2_ activation in the presence of *hPar2 wt* or deletion constructs. While in *hPar2-wt* and *hPar2-K368Z* transfected cells, PAR_2_ activation elicits markedly elevated luciferase Lef/Tcf activity, in *hPar2-K356Z* and *hPar2-truncated* cells PAR_2_ activation failed to increase luciferase Lef/Tcf activity above the basal level. The results were evaluated using GraphPad InStat software and found to be statistically significant (p<0.01). **(E)** Scheme of PAR_2_ C-tail. Schematic representation of PAR_2_ C-tail and its various deleted constructs.

While previously we have demonstrated a novel link between PAR_1_ and β-catenin stabilization [25–27], the role of PAR_2_ in the dynamics of β-catenin stability is unknown. We chose to focus on RKO cells, a colorectal transformed cell line that exhibits intact β-catenin signaling machinery, transformed on a background of microsatellite instability (which leads to hypermethylation of the hMLH1 promoter). RKO cells are of *wt* APC as well as intact β-catenin and p53 machinery system (frequently used for studies of Wnt/β-catenin pathway). Pretreatment (i.e., 2h) with 40mM LiCl (a known GSK3β inhibitor) followed by activation of PAR_2_ induced an increase in β-catenin levels (Figure 1A). Moreover, in HEK293T cells transiently transfected with *hPar2-wt* and *flg*-β-catenin, the SLIGKV activation of PAR_2_ leads to nuclear localization and accumulation of β-catenin (Figure 1B). SLIGKV PAR_2_ activation induces also β-catenin transcriptional activity as shown by the elevated Lef/Tcf luciferase activity (Figure 1D). Therefore, in the presence of a full length *hPar2* construct, a significantly marked increase in the level of β-catenin and its transcriptional activity was observed (Figure 1D).

We next set out to determine the minimal PAR_2_ C-tail region that is required for β-catenin stabilization. For this purpose, several randomly generated deleted *hPar2* C-tail plasmids (e.g. *hPar2* K390Z, *hPar2* K378Z, *hPar2* K368Z, *hPar2* K356Z and truncated *hPar2* that was devoid the entire cytoplasmic tail) were prepared and analyzed for their ability to elicit β-catenin stabilization. Deletion constructs were obtained through substituting amino acids at various sites along the cytoplasmic tail with a premature stop codon. When HEK293T cells were transiently transfected with each of the *hPar2* constructs (including *wt*), as well as with a *flg*-β-catenin plasmid, the following outcome was obtained. After PAR_2_ SLIGKV activation, cell lysates were prepared and Western blot detection was carried out using anti-*flg* antibodies. In contrast to PAR_2_-*wt*, the truncated plasmid as also the shortest C-tail, *hPar2* K356Z, both were unable to induce β-catenin stabilization (Figure 1C), whereas the other various deleted *hPar2* constructs effectively elicited marked levels of β-catenin. Since deletion of the K356-K368 region potently abrogates PAR_2_-induced β-catenin stabilization, we conclude that this region, e.g. the sequence corresponding to K356-K368 NH_2_-NALLCRSVRTV-COOH, is critical for the function of the PAR_2_-enhanced β-catenin signaling pathway. Residues AKNALLCRSVTV were previously proposed as the Ca++signaling site (38). We did not address here whether Ca^++^ signaling is a prerequisite for PAR_2_ induced β-catenin stabilization, and presently it is an open question. However, when we applied the established Ca^++^ blocker; BAPTA to our PAR_2_ induced β-catenin stabilization system, a marked inhibition was seen (Supplementary Figure 1). When we compared the potency of SLIGKV versus trypsin in PAR_2_ induced β-catenin stabilization, the following outcome was observed. While a small increase in β-catenin level is seen following SLIGKV application as soon as 30 min, a marked increase was observed by 4 and 5 hr activation (Supplementary Figure 2). Trypsin showed an increase only after 4 and 5 hr activation (Supplementary Figure 2).

We next assessed PAR_2_-induced β-catenin transcription activities, and determined the minimal PAR_2_ C-tail region that is required for this activity. Co-transfection of RKO cells with the TOPflash-luciferase construct (TOP, TCF optimal promoter containing three copies of LEF-1 binding sites) and with *hPar2-wt, hPar2-K368Z, hPar2-K356Z* or *hPar2-truncated* constructs, was carried out. As shown in Figure 1D, elicited Lef/Tcf promoter activity was seen following PAR_2_ activation when either *hPar2-wt* or *hPar2-K368Z* were present. In contrast, in the presence of either *hPar2-truncated* or the shortest C-tail, *hPar2-K356Z* plasmids, low levels of luciferase promoter activity were observed, similar to control, nonactivated cells. We thus confirm that the sequence NH_2_-NALLCRSVRTV-COOH of PAR_2_K356-K368 C-tail is required for β-catenin transcriptional activity, in agreement with the above described β-catenin stabilization data. Hence, the assigned PAR_2_ C-tail; K356-K368 was identified as necessary for PAR_2_ enhanced β-catenin stabilization and transcriptional activity.

### LRP6 acts as a coreceptor with PAR_2_

**Figure 2:**
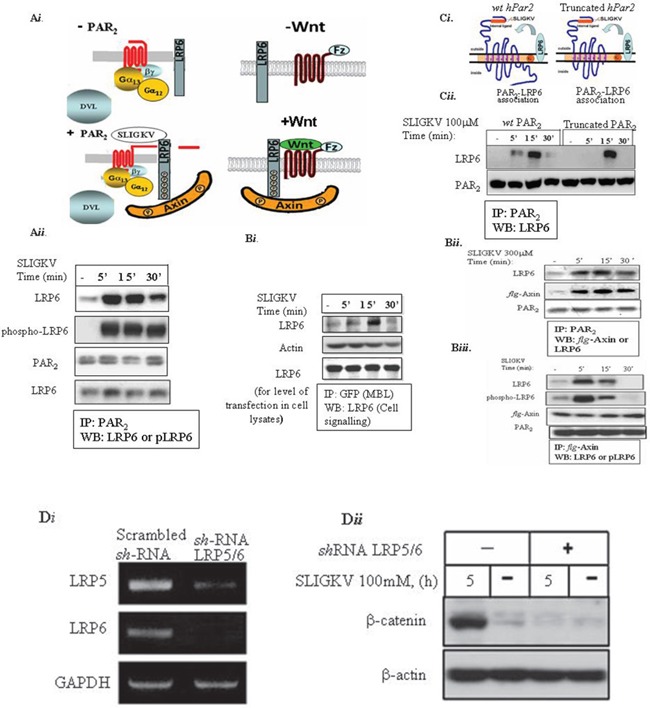
LRP6 is a coreceptor of PAR2 **(Ai)** A scheme of PAR_2_ and co-receptor LRP6 **(A*ii***) HEK293T cells were transiently transfected with HA-*Lrp6, wt hPar2* and *flg*-Axin. Following SLIGKV PAR_2_ activation, co-immunoprecipitation assay was carried out using anti-PAR_2_ abs, and LRP6 was detected following application of anti-HA (LRP6) antibodies. As early as 5 min following PAR_2_ activation and for up to 30 min, PAR_2_ and LRP6 were shown to be within the same immune complex. LRP6 phosphorylation was detected by anti-phospho-LRP6 abs. (**A*iii***) HEK293T cells were transiently transfected with HA-*Lrp6* and *YFP- hPar2*. Following SLIGKV PAR_2_ activation, co-immunoprecipitation assay was carried out using anti-GFP abs (MBL), and LRP6 was detected following application of anti-HA (LRP6) antibodies. (**B*i***) Axin is present within the immune-complex of PAR_2_ and LRP6. Immunoprecipitation using anti-PAR_2_ abs shows distinct co-association with Axin as detected by anti-*flg* antibody application. **(B*ii***) Axin is present in a complex with LRP6 following SLIGKV PAR_2_ activation. Immunoprecipitation analysis of HEK293T cells following PAR_2_ SLIGKV activation was carried out using anti-*flg*-Axin. Detection by either anti-phospho-LRP6 abs or anti-LRP6 showed a profound presence within the same immune complex of LRP6 and Axin. **(C*i***) A truncated form of PAR_2_ associates with LRP6. A scheme representing PAR_2_ and the coreceptor LRP6 association. **(C*ii***) HEK293T cells were transiently transfected with *Lrp6, wt hPar2*, and truncated *hPar2*. Following SLIGKV PAR_2_ activation, co-immunoprecipitation was carried out using anti-PAR_2_ abs and LRP6 was detected by application of anti-LRP6. For 5 min following PAR_2_ activation and for up to 30 min, PAR_2_ and LRP6 were found within the same immune complex. This binding association also occurred when a truncated *hPar2* was used. **(D*i***) *shRNA*-LRP5/6 inhibits effectively LRP5&6 levels. Total cell RNA isolated from HEK-293T cells that were infected either with *sh*RNA-LRP5/6 or nonspecific scrambled *sh*RNA was isolated and RT-PCR analysis was performed using primers for LRP5, LRP6, and GAPDH (used as the internal control). *sh*RNA-LRP5/6 effectively inhibited LRP5/6 levels. **(D*ii***) *sh*RNA-LRP5/6 inhibits PAR_2_-induced β-catenin stabilization. HEK-293T cells were transfected with *flg*-β-catenin and *hPar2-wt* following infection with *sh*RNA-LRP5/6 viral vectors. Next, the cells were activated with SLIGKV (100μM, 5hr). Lysates were prepared and immunoblots were detected using both anti-*flg* (for *flg*-β-catenin) and anti-β-actin as a control for protein loading. The *sh*RNA constructs significantly inhibited PAR_2_-induced β-catenin stabilization.

Both LDL-receptor-related proteins 5 and 6; LRP5 and LRP6 are key components for the activation of β-catenin signaling in the canonical Wnt signaling pathway. It should be noted that while LRP5 and LRP6 exhibit high homology, they may not be equivalent in their ability to transduce Wnt signals. LRP6 independently induces axis duplication in Xenopus embryos, whereas LRP5 does not [28]. LRP6 knockout in mice is embryonically lethal, whereas LRP5-deficient mice are viable and fertile [29]. We then analyzed the possible involvement of LRP6 in PAR_2_-induced β-catenin signaling. We first asked whether LRP6 is recruited to PAR_2_ following SLIGKV activation. Application of anti-PAR_2_ antibodies to HEK293T cell lysates that were transfected with *lrp6* and *hPar2* plasmids showed unequivocally the presence of *lrp6* within the same immune-complex of PAR_2_ (Figure 2Aii). When immunoprecipitation was carried out using YFP-*hPar2* plasmid cotransfected with *lrp6* and detected by application of anti GFP antibodies, a similar result was obtained (Figure 2Aiii). LRP6 is distinctly seen within PAR_2_ immunocomplex following SLIGKV activation mainly after 10 min. When the phosphorylation status of the recruited LRP6 was assessed, it showed the presence of phosphorylated LRP6 (Figure 2Aii&2Bii). Hence, LRP6 is a coreceptor of PAR_2_ present functionally active within one immunocomplex following PAR_2_ activation. Next, the possibility that Axin is recruited to PAR_2_ immunocomplex following SLIGKV PAR_2_ activation was addressed. HEK293T cells were transiently transfected with *hPar2, lrp6* and *flg*-Axin plasmids followed by co-immunoprecipitation analyses. Indeed, a significant level of Axin was found within PAR_2_ immunocomplexes following SLIGKV activation (Figure 2Bi&ii). Hence, Axin is actively engaged to the cell membrane following PAR_2_ activation (bound to pLRP6), forming PAR_2_-LRP6-Axin axis. It appears that the association between PAR_2_ and LRP6 takes place using the extracellular portion of PAR_2_, since also in the presence of a truncated form PAR_2_ lacking its entire cytoplasmic tail, the PAR_2_-LRP6 complex is formed (Figure 2Ci&ii).

We next examined whether silencing LRP5/6 would affect PAR_2_-induced β-catenin stabilization. This was carried out by introducing a mixture of *sh*RNA pool of silenced *lrp5* as also silenced *lrp6* (e.g., *sh*LRP5/6) that effectively silenced both *lrp5* and *6* (see Figure 2Di). Knocked-down *lrp5/6* markedly attenuated PAR_2_-induced β-catenin stabilization (Figure 2Dii), indicating that LRP6 is a critical mediator of PAR_2_-induced β-catenin stabilization.

**Figure 3:**
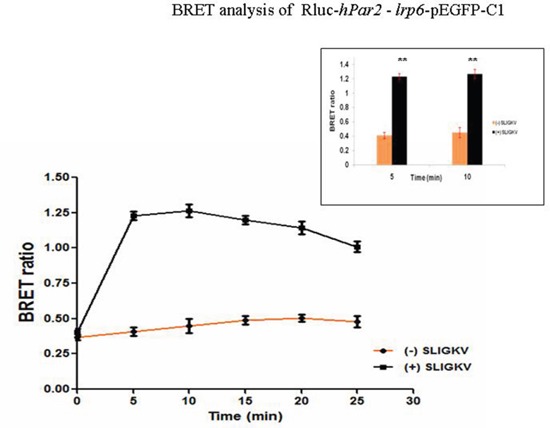
BRET assay shows specific PAR2-LRP6 interaction in fibrocystic HU cells The existence of PAR_2_-LRP6 receptor-receptor interaction and SLIGKV agonist regulation is shown by quantitative BRET^2^ assay in fibrocystic HU cells. HU cells were co-transfected with Rluc-*hPar2* and *lrp6*-pEGFP-C1. Specific maximal signal emission is detected beginning 5 min following SLIGKV activation (*P*=0.002) for up to 30 min, as compared with the non-activated status. This is a representative curve of three independent experiments performed.

We have further confirmed the co-association between PAR_2_ and LRP6 using BRET^2^. BRET has been successfully applied to study receptor-receptor interactions, serving as a powerful tool to assess the nature of these interactions at the molecular level. BRET^2^ assays use the combination of luciferase from *Renilla reniformis* (Rluc) and GFP variants from *Aequorea victoria*, which do not interact without a specific trigger, thus limiting non-specific and random associations. A resonance energy transfer and signal emission from the acceptor partner take place if the distance between the two interacting components is less than approximately10 nm [30, 31]. Not only the distance between these components is a limiting factor, but also the extent of the overlap between the emission spectrum of the donor and excitation of the acceptor [30]. When we transfected HU cells with Rluc-*hPar2* and *lrp6*-pEGFP-C1 constructs following SLIGKV PAR_2_ activation, the BRET ratio revealed that in comparison with the inactive status of PAR_2_, activated PAR_2_ is found in a close proximity with the fluorescent-tagged LRP6. This receptor-receptor interaction further induces a measurable energy transfer in the form of a BRET signal. As shown in Figure 3, in a PAR_2_-LRP6 co-transfected cells, SLIGKV peptide activationfor 5 min induces a highly significant (*p*=0.002) maximal increment in BRET ratio. This signal declines in a time dependent manner, showing the lowest difference at 30 min of SLIGKV activation (Figure 3).

**Figure 4:**
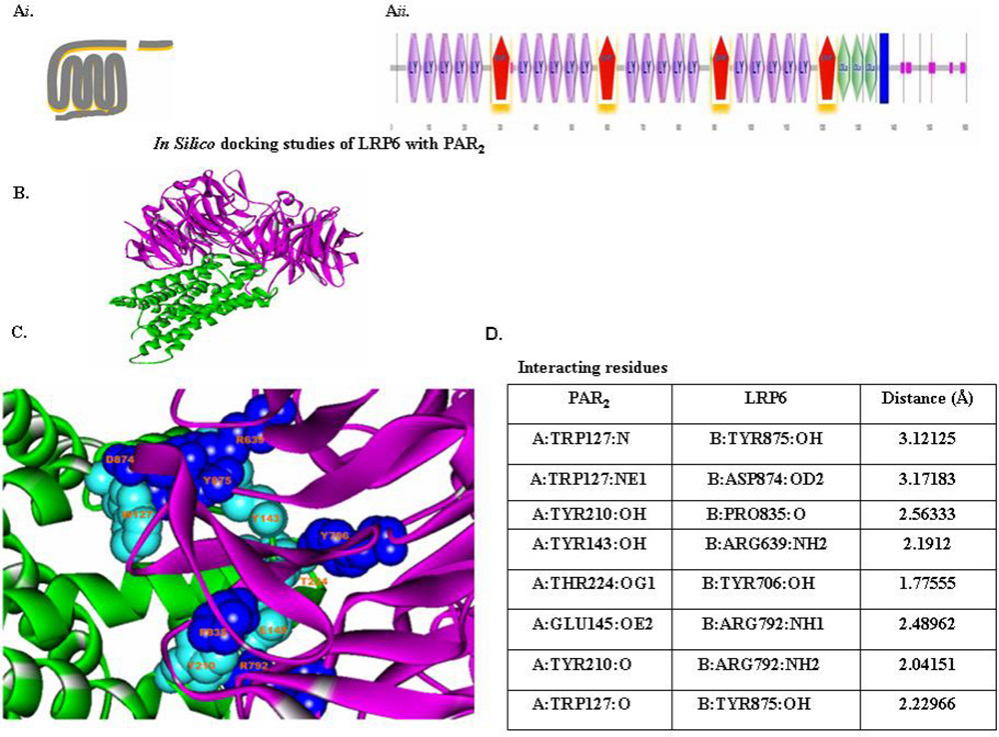
In-silico docking studies of LRP6 with PAR2 (**A*i***) Schematic presentation of PAR2. (**A*ii***) Schematic presentation of LRP6. The domains present in LRP6 were predicted by Bioinformatics Tool SMART (Simple Modular Architecture Research Tool), an online resource (http://smart.embl.de/) that allows identification and annotation of genetically mobile domains and analysis of domain architecture. LRP6, a member of LDLR family, consists of four EGF domains and three LDL repeats (LDLR). **(B)** Protein-protein docking study. Interaction between LRP6 (magenta) with PAR_2_ (green) were determined with the ZDOCK protein–protein docking server, based on a Fourier transform method to search all possible binding modes for the applied proteins. Finally, most probable predictions were ranked on the basis on geometry, hydrophobicity, and electrostatic complementarity of the molecular surface using ZDOCK. **(C)** Determination of interacting residues (e.g., LRP6 with PAR_2_). The interacting residues are shown in CPK (Corey-Pauling-Koltun), a ball-shaped interacting amino acid residue, with cyan CPK for PAR_2_ and blue CPK for LRP6. **(D)** List of PAR_2_ and LRP6 interacting residues.

ZDOCK *protein-protein* docking server information also showed the association between PAR_2_ and LRP6. The protein domains are predicted by Bioinformatics Tool SMART (Simple Modular Architecture Research Tool), an online resource (http://smart.embl.de/) that allows identification and annotation of mobile domains and analysis of the domain architecture (Figure 4).

**Figure 5:**
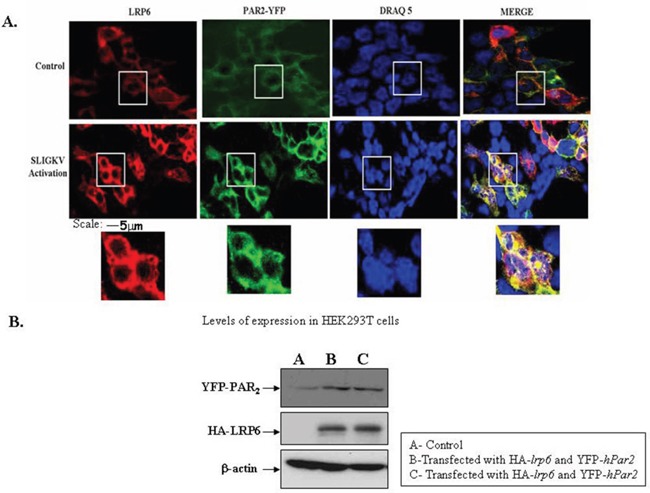
SLIGKV PAR2 activation induces colocalization between LRP6 and PAR2: confocal imaging **(A)** Confocal immunofluorescence analysis. HEK293T cells were transiently transfected with HA-*lrp6*, YFP- *hPar2* and activated with SLIGKV. PAR_2_ was visualized by direct fluorescence (green) and LRP6 by anti-HA antibodies followed by Cy3-conjugated IgG secondary antibodies. For reference, staining of cell nuclei with DRAQ5 (blue) is shown. Merge staining of both LRP6 and PAR_2_ co-localization is shown (yellow), following SLIGKV PAR_2_ activation. **(B)** Western blot analysis indicates levels of YFP-PAR_2_ and HA-LRP6 following transfection. β-actin served as a reference for loading. Data shown are representative of four independent experiments.

The association between PAR_2_ and LRP6 was also assessed by *in situ* confocal microscopy. For this purpose, HEK293T cells were transiently transfected with both HA-*lrp6* and YFP-*hPar2*. After 24 hrs, the cells were serum deprived for additional 24 hrs then activated with SLIGKV for 15 min. PAR_2_ was visualized by fluorescence (e.g., green) level and LRP6 by anti-HA antibodies followed by Cy3-conjugated IgG secondary antibodies (e.g., red; Figure 5A). SLIGKV activation resulted in a merged yellow staining, indicative of co-localization between PAR_2_ and LRP6 (Figure 5A). This result is obtained under conditions whereby equal levels of either YFP-PAR_2_ or HA-*lrp6* in the HEK293T cells were observed (Figure 5B).

### PAR_2_ induces mammary gland tumors

**Figure 6:**
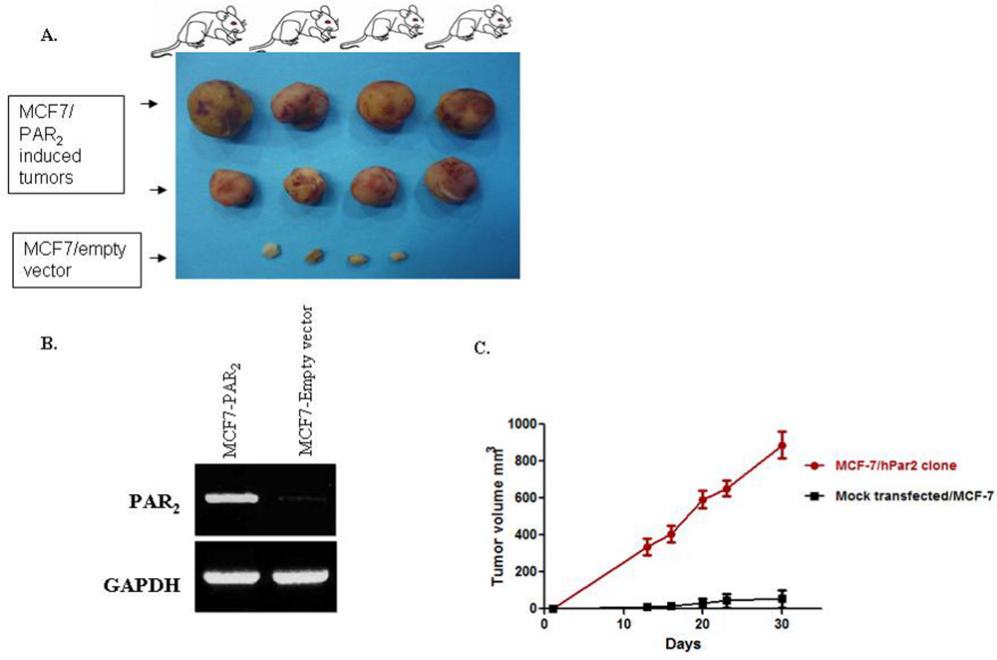
PAR2 elicits mammary gland tumors in a xenograft mouse model **(A)** Morphological appearance of PAR_2_-induced mammary gland tumors shows large and vascularized tumors obtained by orthotopic inoculation of MCF-7 clones stably overexpressing *hPar2* (MCF7/*hPar2*), as compared with mock-transfected clones (MCF7/empty vector). **(B)** RT-PCR analyses demonstrating the levels of *hPar2* in the stable clones, as compared with a house-keeping gene GAPDH. **(C)** Tumor volume of the two groups is shown. Error bars show s.d.; *P < 0.006. Data shown are representative of three independent experiments.

Stable cell clones (e.g., MCF-7 cells) overexpressing PAR_2_ were assessed for their capabilities to generate orthotopic mammary fad pat tumors. Following subcutaneous implantation of slow release pellets of β-estradiol (Innovative Research of America, Sarasota, FL, USA), MCF-7 clones were inoculated in the mammary fat pad with MCF7/*hPar2* stable clones. After appropriate periods of time the PAR_2_ overexpressing clones elicited large mammary gland tumors, as compared with control mock-transfected mice, which generated very small tumors (Figure 6A&6B). Tumor volume growth of the PAR_2_ instigated tumors as compared with control mock transfected cells was evaluated (Figure 6C). RT-PCR analysis confirmed that the MCF-7 clones expressed PAR_2_, in contrast with the mock transfected cells, which were lacking PAR_2_ (Figure 6B).

**Figure 7:**
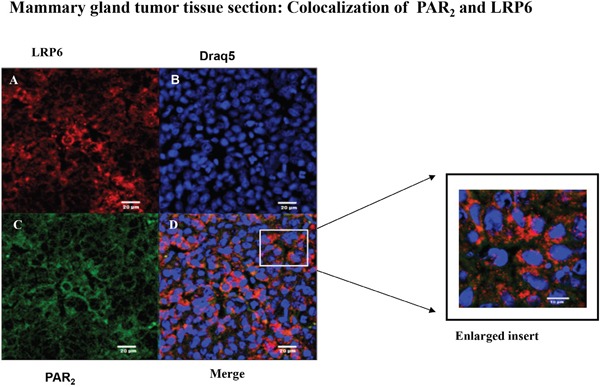
Colocalization between LRP6 and PAR2 in breast mouse cancer tumor biopsies: confocal analysis Confocal immunohistostaining was carried out on PAR_2_-induced mouse orthotopic mammary gland tumors. Application of anti-LRP6 (**A**; red) and anti-PAR_2_ (**C**; green) Abs was followed by appropriate Cy2- and Cy3-conjugated IgG secondary antibodies. The cell nuclei were visualized by DRAQ5 (**B**; blue). High expression levels were observed in both LRP6 and PAR_2_. Merge analyses indicate co-localization between LRP6 and PAR_2_ (**D**; orange). Data shown are representative of three independent experiment. Magnification x40.

In order to substantiate *in vivo* the PAR_2_ and LRP6 co-association that was observed *in vitro*, we have analyzed PAR_2_-driven mammary gland tumor tissue sections by confocal staining for the colocalization between PAR_2_ and LRP6. Toward this goal, tissues were double-stained for LRP6 (A; red) and PAR_2_ (C; green). Abundant distribution of either LRP6 or PAR_2_ was seen in the large mammary gland tumor sections (Figure 7A&7C). Merge analysis indicated colocalization of PAR_2_ with LRP6, as shown by the yellow staining (Figure 7D). Inset is a magnified view of the boxed area. It is postulated that the tumors exhibit plentiful protease in the vicinity of the tumor microenvironment, sufficient to continuously activate PAR_2_. As a result, colocalization with LRP6 is observed during PAR_2_ induction and initiation of large tumor formation, *in vivo*.

### Both Gα_13_ and Gα_12_ are involved in PAR_2_-mediated β-catenin stabilization

**Figure 8:**
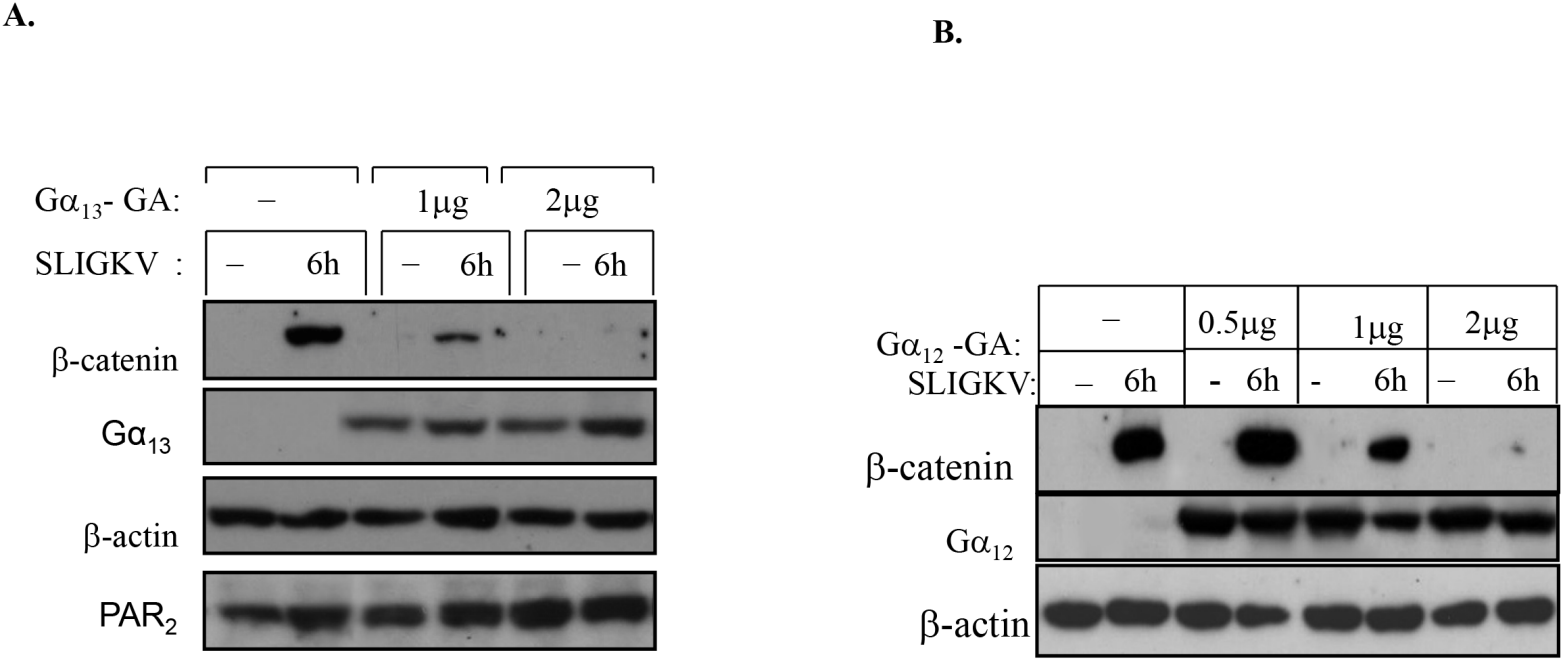
Both Gα13 and Gα12 are involved in PAR2-induced β-catenin stabilization **(A)** A dominant-negative (DN) form of Gα13 inhibits β-catenin stabilization. HEK-293T cells were transiently transfected with *hPar2-wt, flg*-β-catenin, empty vector, or dominant-negative Gα_13_-GA plasmid. Following PAR_2_ activation, immunoblots were analyzed using anti-*flg* (for *flg*-β-catenin), anti-Gα_13_, anti-PAR_2_, and anti-β-actin antibodies. Gα_13_ potently inhibited PAR_2_-induced β-catenin stabilization in a dose-dependent manner. **(B)** A DN form of Gα_12_ inhibits β-catenin stabilization. HEK-293T cells were transiently transfected with *hPar2-wt, flg*-β-catenin, empty vector, or dominant-negative Gα_12_-GA plasmid. Following PAR_2_ activation, immunoblots were analyzed using anti-*flg* (for *flg*-β-catenin), anti- Gα_12_, and anti-β-actin antibodies. Gα_12_ as well as Gα_13_ potently inhibited PAR_2_-induced β-catenin stabilization in a dose-dependent manner. Since both Gα_13_-GA and Gα_12_-GA independently abrogate PAR_2_-induced β-catenin accumulation, there is most likely an overlap between Gα_12_ and Gα_13_ in mediating the induction of β-catenin stabilization.

Since Gα_12/13_ are the only G-proteins known to be involved in cell transformation [32–34], we sought to analyze the relationship between these G-proteins and PAR_2_-induced β-catenin stabilization. In order to dissect their relative involvement, dominant negative (DN) forms of Gα_12_ and Gα_13_ [26, 34] were utilized. When we transiently transfected HEK293T with *flg*-β-catenin, *wt hPar2*, and either a dominant negative Gα_12_-GA or Gα_13_-GA mutants, the following data were obtained. After activation of PAR_2_, cell lysates were prepared and immunoblots were further evaluated. Application of anti-*flg* (to detect levels of β-catenin) or anti-Gα_12_ and Gα_13_ antibodies was carried out. We have found that increased expression of the dominant negative forms of either Gα_13_ or Gα_12_ (i.e., Gα_13_-GA or Gα_12_-GA) both markedly inhibited PAR_2_-induced β-catenin stabilization in a dose-dependent manner (Figure 8A&8B). In contrast, PAR_1_ acts specifically via Gα_13_ [26]. The presence of increasing concentrations of Gα_12_-GA did not show any effect on PAR_1_-induced β-catenin levels, whereas the DN Gα_13_-GA construct effectively inhibited β-catenin levels [26]. Taken together, these findings suggest that there is, most likely, an overlap in PAR_2_-induced β-catenin stabilization with regard to the role played by Gα_12_ and Gα_13_, and both Gα_12_ and Gα_13_ are required to induce PAR_2_-mediated β-catenin stabilization.

### Translocation of DVL to the cell nuclei by PARs

**Figure 9:**
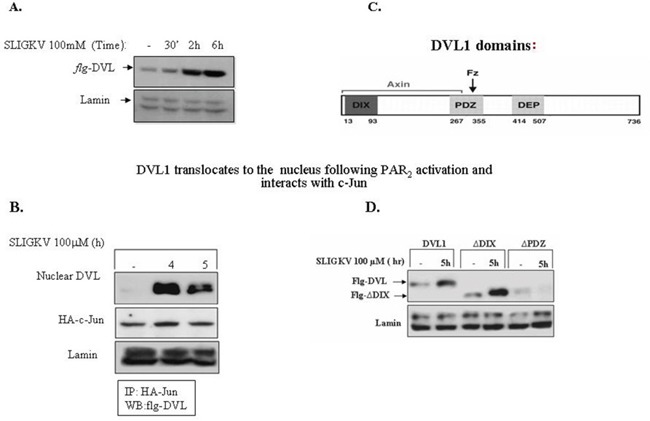
DVL1 translocates to the cell nucleus following PAR2 activation **(A)** DVL1 translocates to the cell nucleus following PAR_2_ activation and PAR_2_ activation. HEK-293T cells were transiently transfected with *flg*-dvl1 and *hPar_2_*. The nuclear fraction was isolated and Western blots were analyzed using anti-flg for the detection of DVL1 and anti-lamin antibodies as loading control. DVL accumulated in the nucleus in response to PAR_2_ activation. **(B)** DVL interacts with c-jun in the nucleus following PAR_2_ activation. PAR_2_ induces the interaction between DVL1 and c-Jun. HEK293T cells transfected with both *flg*-Dvl1 and c-Jun showed nuclear localization of DVL1 as judged by immunoprecipitation analyses. PAR_2_-induced association of DVL with c-Jun occurred in a manner similar to Wnt signaling. **(C)** Scheme of DVL1 domains. **(D)** DVL-PDZ domain is required for PAR_2_-induced DVL1 nuclear localization. HEK-293T cells were transiently transfected with the following constructs: *flg*-*wt* dvl1, DVL1 deletion constructs (e.g., *flg*-ΔDIX-dvl or *flg*-ΔPDZ-dvl), and *hPar_2_*. The nuclear fraction was isolated after PAR_2_ activation and immunoblots were analyzed using anti-*flg* and anti-lamin antibodies. The deleted DVL-PDZ domain construct, but not the DIX domain-deleted construct, failed to translocate to the nucleus, as compared with *wt* DVL nuclear localization. It is concluded that the PDZ, but not the DIX domain, is necessary for PAR_2_-induced nuclear localization of DVL1.

While DVL acts as an upstream cytoplasmic link initiating the process of β-catenin stabilization, it is now well recognized that DVL also exists in the cell nucleus, where it is required for the Wnt-β-catenin transcriptional activity eliciting downstream targeted gene expression. This renders DVL a more complex role than initially thought. In addition to serving as a scaffolding protein bridging seven transmembrane receptors and signaling components [35–37], it also acts as part of a nuclear transcription complex. At least in the case of PAR_1_ [25, 26], as also the parathyroid receptor1 (PTH1R), DVL acts as a scaffold bridge, linking to GPCRs and consequently enabling the *early-on* event in β-catenin stabilization [39]. We demonstrate now that following SLIGKV PAR_2_ activation DVL is ultimately found within the cell nucleus as soon as 30 min after activation and distinctly by 2hr (Figure 9A). Furthermore, we show here that DVL and c-Jun form a complex within the cell nuclei. This takes place following transient transfection of *flg-dvl*, HA-c-*Jun* and *hPar_2_* plasmids in HEK293T cells. Immunoprecipitation using anti-HA antibodies and *flg*, Western blot detection showed a noticeable complex formation with DVL and c-Jun, in the cell nuclei, by SLIGKV PAR_2_ activation that became prominent after 5h PAR_2_ activation (Figure 9B). Indeed, it was previously reported that at a later time period and upon Wnt signaling, DVL translocates to the cell nucleus where it forms a transcription complex among others, with nuclear β-catenin and c-Jun [39]. Similarly, nuclear translocation of DVL was found also following activation of PAR_1_ (see Supplementary Figures 3&4).

DVL protein comprises an N-terminal DIX domain, a central PDZ domain, and a C-terminal DEP domain (see scheme, Figure 9C). Wewished to identify which of these regions are essential for DVL nuclear localization. Toward this purpose, we performed Western blot analyses from nuclear extracts of cells expressing *dvl* constructs of either *wt* full length or the deleted domains of DIX or PDZ-(deleted for DIX or for PDZ). Transfections with *flg*-*wt*-*dvl, flg*-*dvl*-ΔDIX, or *flg*-*dvl*-ΔPDZ showed that efficient translocation of DVL is found after 5 hr of PAR_2_ activation using the DIX deleted construct, similar to the *wt* DVL (Figure 9D). In contrast, nuclear localization of DVL was completely abrogated in the presence of the PDZ deleted form (Figure 9D). This suggests that PDZ domain is required for DVL nuclear localization. A similar outcome is demonstrated for PAR_1_ DVL nuclear localization (see Supplementary Figure 5). This stands in contrast to the *early-on* DVL binding to membrane-anchored PAR_1_, whereby the DIX domain was found to be essential for the PAR_1_-Gα_13_ axis formation [26]. In accordance with the involvement of both Gα12 and Gα13 in PAR_2_-induced β-catenin stabilization, this also takes place with regard to PAR_2_-induced DVL nuclear localization (Supplementary Figure 6). DVL is necessary also for PAR_1_-induced β-catenin stabilization (Supplementary Figure 7).

## DISCUSSION

Here we show that PAR_2_ is a significant inducer of the β-catenin stabilization path in cancer. We demonstrate the novel recruitment of LRP6 following SLIGKV-PAR_2_ activation, and show for the first time that PAR_2_ associates with LRP6, a known coreceptor of *Frizzled* (Fz) proteins in the Wnt/β-catenin route, following SLIGKV activation. The association is demonstrated using various methodologies, namely co-immunoprecipitation, BRET, and also confocal microscopy image analysis. These assays are supported by the ZDOCK *protein-protein* server, which predicts the tight association between PAR_2_ and LRP6.

PAR_2_ association with LRP6 takes place via the interaction of the PAR_2_ extracellular portion on the cell surface. As a result, LRP6 is phosphorylated and further binds Axin, which is being dislodged from the “degradation complex”, ultimately leading to β-catenin stabilization. Taken together, our data suggest a novel path of PAR_2_-induced β-catenin stabilization. We show that the shortest PAR_2_ C-tail capable of eliciting β-catenin stabilization and transcriptional activity is PAR_2_-K368Z, which includes the amino acid sequence “AKNALLCRSVTV,” a Ca^++^ binding domain [38]. It is possible that a PAR_2_-induced Ca^++^ signal is required for β-catenin stabilization, however this remains to be studied. This possibility is raised by our preliminary observations indicating that in the presence of BAPTA (30μM), a known Ca^++^ inhibitor, PAR_2_-induced β-catenin stabilization and transcriptional activity are inhibited (Supplementary Figure 1). Previously, we have demonstrated a direct link between PAR_1_ and β-catenin stabilization both in a transgenic (*tg*) mouse model, overexpressing *hPar1* in the mammary glands and in a spectrum of transformed epithelial cell lines [25–27]. This was shown to be mediated selectively via the association of Gα_13_ and the DIX-DVL axis, formed *early on* to facilitate β-catenin stabilization. PAR_2_-mediated dynamics of β-catenin hyperactivity takes place in a different manner. We now show the equal involvement of both Gα_12_ and Gα_13_ G-proteins in PAR_2_-induced β-catenin stabilization, and the recruitment of Axin to the PAR_2_-LRP6 axis. It is demonstrated that *sh*RNA silencing of LRP6 effectively attenuates PAR_2_-induced β-catenin stabilization; thus, SLIGKV/PAR_2_-induced recruitment of LRP6 is essential for the PAR induced β-catenin signaling. As a result, DVL is translocated to the cell nucleus where it initiates a transcriptional switch, recruiting various chromatin modifiers to β-catenin, among of which are; c-Jun, and Lef/Tcf family members, consequently leading to the transcription of downstream target genes. While DVL-DIX domain is essential for the formation of PAR_1_-Gα_13_-DVL complex, the PDZ domain of DVL is involved in PAR_1_- and PAR_2_-induced DVL nuclear translocation.

LRP6 is a well-recognized coreceptor for Wnt signaling in cancer [40–47]. Whereas Wnt is known to function through the classical *Fz*-LRP6 shared interaction for β-catenin dynamics, our data introduce a new partner for LRP6 association that potently instigates β-catenin stabilization. Similarly, LRP6 was previously shown to act as a coreceptor with another GPCR, the parathyroid receptor1, PTH1R [39]. The PTH hormone activates β-catenin signaling in osteoclasts by the direct recruitment of LRP6 to the PTH/PTH1R complex [36]. This takes place via the extracellular portion of LRP6, that acts with PTH1R, leading to the phosphorylation of the PPPSP motif of the LRP6 cytoplasmic tail [48]. Interestingly, PTH1R was found also capable of recruiting DVL to the PTHR1 C-tail following PTH activation [39], as we previously demonstrated for PAR_1_ [26].

LRP5 and LRP6 are type I, single-span transmembrane receptors with a large extracellular domain, that were shown to bind several Wnt ligand species shown *in vitro* experiments [28, 45–47]. In addition to Wnt proteins, the extracellular portions of LRP5 and LRP6 also bind other agonists and antagonists of the Wnt pathway, such as DKK1, Sclerostin, and Wise, members of the Dkk family [46, 48–51]. LRP5 and LRP6 exhibit a high degree of sequence homology, sharing 73% and 64% sequence identity in their extracellular and intracellular domains, respectively [35]. The ectodomain of LRP5/6 is composed of four propeller/epidermal growth factor (EGF) repeats (E1-4), three LDL repeats (LDLR) and five intracellular PPP(S/T)P domains that mediate downstream signaling events [47]. E1-4 but not LDLR is the binding domain of canonical Wnt ligands and the canonical pathway inhibitor Dkk [45, 46, 48, 51]. Until now, the LDLR-binding proteins remain unexplored. The current view is that the close proximity of LRP5/6 and *Fz* coupled by canonical Wnt ligand binding to E1-4 of LRP5/6 and amino-terminal cysteine-rich domain (CRD) of *Fz* is needed for canonical pathway activation [45, 49]. In contrast, Dkk1 promotes the internalization of LRP5/6 via binding with Dkk1 receptor Kremen, making LRP5/6 unavailable for Wnt reception and inhibiting the canonical pathway [50].

This, coupled with extensive similarities in structural and biochemical properties, has led to the assumption of functional redundancy between the two receptors; however, *in vivo* studies show that they mediate unique functions. In addition, homozygous deletion of LRP6 in transgenic mice leads to perinatal lethality, while LRP5 knock-out mice are viable and fertile [29, 52, 53]. Recently however, it was found that LRP5-deficient mice develop low bone mass postnatally [28, 54–56], which was attributed to a direct effect of LRP5 on osteoblast function. Nevertheless, the role of LRP5 in Wnt signaling remains unknown, since the absence of LRP5 has no effect on Wnt3a-mediated transactivation of the canonical Wnt pathway in *lrp5*^_/_^ mammary epithelial cells (MECs) [57].

Recently, it was proposed that while LRP5/6 and *Fz* are oncogenic in nature, through direct binding (Fz and LRP5/6) they are able to prevent *Fz*-regulated non-canonical pathway activation and the non-canonical mediated tumor metastasis. Knocking down endogenous LRP5/6 promoted otherwise nonmetastatic tumor cells to disseminate throughout the body. Along this line of evidence, the application of soluble recombinant LRP6 extracellular domain effectively inhibited the appearance of metastatic foci from otherwise aggressive metastatic tumor cells. Hence, according to these findings, therapeutic medicaments based on anti-LRP6 should be posibly reconsidered since they may induce metastatic spread [58].

DIX domain is in fact found in three proteins, Axin, DVL, and Ccd1. A previous study suggested that DVL may behave as a dominant-negative protein of Axin, regulating its function via the heterotypic interaction between DVL-DIX and Axin-DAX (disheveled and Axin domains) [59]. The Axin DAX domain mediates homo- and heteropolymerization, which may be important for its function [59–61]. Previous studies have suggested that the Axin N-terminus portion, including the RGS domain and the linker region between the RGS domain and the GSK-binding domain, has an inhibitory role on Axin's binding with its partners [63, 64]; however, the mechanism underlying this inhibition remains elusive [65]. Two recent publications propose a model in which Axin is regulated via conformational change of an “open” or activated conformation as well as a “closed” or auto-inhibited structure. Axin is a phosphoprotein that is central to assemblies of both destruction [66–69] and signaling complexes [28, 51, 62, 70, 71], and it becomes dephosphorylated upon Wnt stimulation [72]. Kim *et al*., [65] proposed that without Wnt, Axin is associated with and phosphorylated by GSK3β, which is present in an activated (“open”) conformation for β-catenin binding and is poised for engagement of LRP6. In the presence of Wnt, LRP6 undergoes *Fz*/DVL-dependent phosphorylation and recruits active Axin from the “destruction complex” to form the signaling queue, in which GSK3β bound to Axin is inhibited by the phospho-LRP6 [73–76], consequently leading to inhibition of β-catenin phosphorylation which tips the balance toward Axin dephosphorylation by PP1 phosphatase. The dephosphorylated Axin form adopts an inactivated (“closed”) conformation through intramolecular auto-inhibition and becomes incompetent for association with β-catenin or phospho-LRP6, leading to the disassembly of destruction and signaling complexes. Similarly, it was suggested by the group of Lin Li [77] that auto-inhibitory conformation of Axin is mediated by interactions between its N- and C-terminal domains. It was shown that conformational change is most likely regulated by the Wnt signaling pathway and is further facilitated by HLY78, a small molecule that binds Axin and activates Wnt signaling. It has been postulated that the direct binding of HLY78 to the DAX domain of Axin triggers the conformational change of Axin from a ‘closed' auto-inhibitory state to an ‘open’ active state, leading to enhancement of the Axin-LRP6 association and the subsequent phosphorylation and activation of LRP6. Hence, these two independent publications strongly support and strengthen the conformation change scenario in Axin as a key regulatory change controlling Wnt signaling. We find that SLIGKV activation of PAR_2_ leads to LRP6 recruitment followed by the binding association of the Axin, most likely similar to Wnt activation, yet to be fully described.

Formation of the β-catenin-TCFs complex has been well established as a prerequisite for c-Jun but not of endogenous c-Fos, indicating that c-Fos did not participate in the canonical Wnt signaling as did c-Jun, and knocking down endogenous c-Jun with *sh*RNA markedly suppressed Wnt-3a-induced transcriptional activity in HEK293T cells [75]. In-parallel, the expression of *c-myc* target gene failed to respond to canonical Wnt stimulation in *c-Jun* −/−cells [77].

In zebra fish, loss of c-Jun function inhibited the induction of ventral mesoderm and reduced the expression of ventral marker genes in a manner that was very similar to the phenotype obtained by the loss of function of *Wnt-8*. Two previous studies have demonstrated that DVL, a pivotal regulator of the canonical Wnt pathway, is also localized in the nucleus [35, 37], and that its nuclear localization is required for the canonical Wnt signaling [35]. It is well known that β-catenin directly interacts with TCFs *in vitro* and *in vivo*; however, insight as to how these interactions take place has been only recently gained. It was demonstrated [78] that DVL can be recruited in the nucleus to the promoter of Wnt target genes interacting with c-Jun and β-catenin, respectively, further enhancing its association with β-catenin-TCFs transcriptional complex.

Nuclear DVL is suggested to be crucial for the formation of a stable complex between β-catenin and TCFs in mammalian cells and zebrafish [78]. We hereby demonstrate that, similar to Wnt signaling, PAR_1_ (or PAR_2_) activation leads to β-catenin-DVL complex formation in the nucleus but not in the cytoplasmic compartment (Supplementary Figure 4). Nuclear DVL is essential for Lef/Tcf transcriptional activity and downstream target gene expression, since *sh*RNA silencing of *dvl* potently inhibited PAR_1_- (or PAR_2_) induced transcriptional activity (Supplementary Figure 4).

The current model thus suggests that DVL may have multiple roles in the canonical Wnt signaling pathway. Initially, cytoplasmic DVL receives a signal from the plasma membrane, resulting in accumulation of β-catenin in the nucleus. Next, DVL nuclear accumulation is promoted [35, 37], involving DVL activation of JNK in a yet unknown manner to promote c-Jun phosphorylation [77, 78]. In the nucleus, DVL binds to phospho-c-Jun and β-catenin, and promotes the formation of a quaternary functional complex consisting of β-catenin, Lef-1/Tcfs, c-Jun, and DVL. We provide evidence that both PAR_1_ and PAR_2_ act to instigate nuclear localization of DVL and its interaction with both c-Jun and β-catenin, corresponding with the proposed path of the canonical Wnt signaling.

## MATERIALS AND METHODS

### Plasmids and reagents

cDNA encoding mouse *flg*-DVL1, *flg*-ΔDIX-DVL1, *flg*- ΔPDX-DVL1, and myc and *flg*- β-catenin were kindly provided by Dr. Ben-Neria (Hebrew University, Jerusalem). cDNA encoding mouse *flg*-DVL1-DIX and flg-DVL1-PDZ were kindly provided by Lin Li (State Key Laboratory of Molecular Biology, Shanghai Institute for Biological Science, Chinese Academy of Sciences, Shanghai 200031, China). The cDNA encoding G_α12_, G_α13_, and G_αq_ constructs, *wt*, was constitutively active and dominant negative as previously described [26]. The PAR_2_ and PAR_1_ agonists TFLLRN and SLIGKVwere obtained from GeneScript (Piscataway, NJ, USA 08854). Thrombin was obtained from OMRIX Bio Pharmaceutical (Ramat Gan, Israel).

### Cells

HCT116, HT29, HEK293T, HU and RKO cells were grown in 10% FCS-DMEM/RPMI supplemented with 50 U/ml penicillin and streptomycin (GIBCO-BRL, Gaithersburg, MD, USA) and maintained in a humidified incubator with 8% CO_2_ at 37°C.

### Mutation generation

PAR_2_ mutants were generated by site-directed mutagenesis using QuikChange (Stratagene, La Jolla, CA) as previously described [17], Briefly, the procedure utilizes a pcDNA3.1(+) vector containing an insert of *hPar2* coding sequence and two synthetic oligonucleotide primers containing the desired mutations. The oligonucleotide primers, each complementary to opposite strands of the vector, are extended during temperature cycling by *PfuTurbo* DNA polymerase (2.5U per reaction). Incorporation of the oligonucleotide primers generates a mutated plasmid containing staggered nicks. Following temperature cycling, the product is treated with *Dpn*I (10U, 37°C, 1 hr). DNA containing the desired mutations is then transformed into XL1-blue super competent bacteria cellsfollowing plasmid isolation using the PureLink™ Maxiprep Kit (Invitrogen Corporation, Carlsbad, CA, USA). Dideoxy sequencing is performed to confirm insertion of the appropriate mutation. The primers are as follows: *S390Z-* GCTCTTACTCTTCAAGT**TGA**ACCACTGTTAAGACCTCC, *K378Z-*CCCTCACCTCAAAG**TAA**CACTCCAGGAAATCCAGC, *K368Z-* GCCGAAGTGTCCGCACTGTA**TAG**CAGATGCAAGTATCCC, *K356Z-*GGGATCATGCA**TAG**AACGCTCTCCTTTGCCGAAGTGTCCGC, *S348Z-*CGACCCCTTTGTCTATTACTTTGTT**TCA**CATGATTTCAGGG.

### Immunohistological staining

Tissue samples derived from PAR_2_ induced mammary glands were fixed with 4% formaldehyde in PBS, embedded in paraffin, and sectioned (5-μm sections). After deparaffinization and rehydration, the sections were stained with H&E or subjected to immunohistochemistry. For this, the slides were incubated 3% H_2_O_2_ prior to antigen retrieval. Antigen unmasking was carried out heating (20 min) in a microwave oven in 10mM Tris buffer containing 1mM EDTA. After blocking slides were incubated with the following primary antibodies: anti β-catenin (C-2206, Sigma-Aldrich St Louis MO, USA), anti PCNA (sc-56, Santa Cruz Biotechnology, USA dilution 1:200) anti DVL1 (sc7397, Santa Cruz Biotechnology, Dallas Texas, USA; goat polyclonal IgG) or anti CD31 (Dako, Clone JC70A, Carpinteria, CA). Color was developed using the 3,3′-diaminobenzidine (DAB) (Thermo Scientific, Walham, MA, USA) or the Zymed AEC substrate kit (Zymed Laboratories So, San-Francisco, CA, USA), followed by counter staining with Mayer's haematoxylin. Controls without addition of primary antibodies showed low or no background staining in all cases.

### Cell transfections

Cells grown to 80% confluency were transfected with 0.5-2 μg/mL of plasmid DNA in Fugene 6 transfection reagent (Boehringer-Mannheim, Mannheim, Germany) according to the manufacturer's instructions.

### RNA isolation and RT-PCR

RNA was isolated with Tri-Reagent (MRC, Cincinnati, OH, USA) according to the manufacturer's instructions. After reverse transcription of 1 μg total RNA by oligo (dT) priming, cDNA was amplified using Taq DNA polymerase (Promega, Madison, WI, USA). Comparative semi quantitative PCR was performed as follows: GAPDH mRNA was first amplified at a low cycle number. If needed, cDNAs were adjusted to obtain similar intensities for GAPDH signals with all the samples. The adjusted amounts of cDNA were subjected to PCR. The PCR conditions were an initial denaturation at 94°C for 2 min, denaturation at 94°C for 15 sec, annealing for 45 sec at the appropriate temperature and extension for 1 min at 72°C (24–33 cycles of amplification). Aliquots (15 μl) of the amplified cDNA were separated by 1.5% agarose gel electrophoresis. VEGF PCR product was separated on a 2% Nusieve (FMC Rockland, ME, USA, 3:1 agarose gel) and visualized by ethidium bromide staining under ultraviolet light.

### *sh*RNA constructs and lentiviral vector production

To prepare *Si*RNA constructs we used U6 promoter driver and a lentivirus-mediated delivery cassette of *Si*RNA (pLentilox 3.7) specific for the target genes: DVL1, LRP5, and LRP6. For this, a sequence of 19 nucleotides of the target gene coding region was selected for stem-and-loop oligonucleotide *sh*RNA. Appropriate DNA oligonucleotides were synthesized to generate the hairpin structure stem-and-loop *sh*RNA expression cassette. The oligos comprised the following: 19 bases of the target gene coding sequence, the loop sequence linker (9 bases), a reverse complement of 19 bases of the target gene coding region, and a terminator sequence poly T. The sticky end of the *Xho*I site was added to the antisense strand oligos. Both sense and antisense sequences were phosphorylated at the 5′ ends. The sense sequence oligos were annealed to their respective antisense oligos. *Si*RNA cassette sequences were then ligated into pLentilox 3.7 vector (Van Parij Laboratory, Massachusetts Institute of Technology, Cambridge, MA, USA). The sequence of *sh RNA* for the target genes was as follows: AACAAG ATC ACCTTCTCCGAG (DVL1), CATGATCGAGTCGTCCAAC (LRP5), CCGCATG GTG ATT GA TGA (LRP6).

### Western blot analysis

Cells were solubilized in lysis buffer containing 10 mM Tris-HCl (pH 7.4), 150 mM NaCl, 1 mM EDTA, 1% triton X-100, and protease inhibitor cocktail including 5 mg/ml aprotinin, 1 mM phenylmethylsulfonylfluoride, PMSF, and 1 mM Na orthovanadate (Sigma, St. Louis, MO, USA), for 20 min at 4°C. After centrifugation at 10,000 g for 20 min at 4°C, the supernatants were transferred and the protein content was measured. Lysates (50 μg) were loaded on a 10% SDS-PAGE followed by transfer to Immobilon-P membrane (Millipore, Bedford, MA, USA). Membranes were blocked and probed with the appropriate antibodies at a concentration of 1 μg/ml. Anti-β-catenin (Cell signaling 9562), anti PAR_2_ (SAM11 Santa Cruz, Dallas Texas, USA), LRP6 antibodies (Cell signaling (C5C7) rabbit mAb Danver, MA USA), anti DVL1 (mAb; sc 8025, Santa Cruz, Dallas Texas, USA), anti Gα12, anti Gα13 and anti β-actin (Santa Cruz, CA, USA), or anti *flg* antibodies (mAbF316, Sigma clone M2) and for IP OctA -Probe(D-8) sc807 polyclonal Ab (Santa Cruz Biotechnology Inc. Dallas, TX, USA) were suspended in 3% BSA in 10 mM Tris-HCl pH 7.5, 100 mM NaCl and 0.1% Tween-20. After washes, blots were incubated with secondary antibodies conjugated to horseradish-peroxidase. Immunoreactive bands were detected by the enhanced chemiluminescence (ECL) reagent (Pierce, Rockford, IL, USA).

### Nuclear extract

Cells were solubilized in lysis buffer A (10mM HEPES, pH 7.9, 10 mM KCl, 0.1mM EDTA and 1mM DTT), a protease inhibitor cocktail (1:100), 1 mM phenylmethylsulfonylfluoride, PMSF, and 1 mM Na orthovanadate (Sigma) for 15 min at 4°C. After adding 10% NP-40 solution, centrifugation at 10,000 g was performed. The pellet was incubated on ice with buffer C (20 mM HEPES, pH 7.9, 420 mM KCl, 1mM EDTA, and 1mM DTT), a protease inhibitor cocktail, 1 mM PMSF, and 1 mM Na orthovanadate. After centrifugation at 12,000 g for 15 min at 4°C, the supernatants were collected and the protein content was evaluated.

### Immunoprecipitation

Protein cell lysates (400 μg) were used for immunoprecipitation analysis. Anti- PAR_2_, or anti-flg antibodies were added to the cell lysates. After overnight incubation, protein A-Sepharose beads were added to the suspension, which was subsequently rotated at 4°C for 1h. Elution of the reactive proteins was performed by resuspending the beads in protein sample buffer followed by boiling for 5 min. The supernatant was then resolved on a 10% SDS-polyacrylamide gel and treated as indicated above for Western blotting.

### BRET assay

Interaction of PAR_2_ with LRP6 was further validated using bioluminescence resonance energy transfer assay (BRET^2^). In our BRET^2^ experiment, we applied Rluc-*hPar2* (a kind gift of the Trejo J-A Department of Pharmacology, School of Medicine, University of California San Diego, La Jolla, CA, USA) as a donor and *lrp6*- pEGFP-C1 as an acceptor. Toward this purpose, we cloned LRP6 downstream of pEGFP. To prepare the *lrp6*-pEGFP-C1 construct, *lrp6* insert was taken from from *lrp6*-pCS2 plasmid (addgene) using Xba-1 and BamH1 restriction enzymes. Next, the *lrp6* insert was subcloned into pEGFP-C1 vector. The cloned construct was confirmed by sequencing and LRP6 expression was also confimed by Western blot using anti-LRP6 antibody (Cell Signaling).

The entire BRET^2^ experiment was performed using well-defined protocols, with some modifications [30, 31]. Briefly, to perform BRET^2^, after concentration optimization, 0.5 μg of Rluc-*hPar2* alone or together with 1 μg of *lrp6*-pEGFP-C1 was transfected into HU cells. The day following transfection, cells were detached and re-seeded (50,000 cells/well) into white opaque 96-well microplates (Thermo Fisher, Waltham, MA, USA). The next day, experimental cells were activated with the PAR_2_ ligand, SLIGKV (200 μM), washed (PBS containing 0.1% D-glucose and 0.5mM MgCl_2_), and immediately after addition of the cell-permeable substrate DeepBlueC^TM^ (10 μM), luminescence was measured using the Tecan Spark^TM^10M multimode microplate detection system. The detection system is equipped with two distinct filters. Here, filter 1 (410 ± 70nm) was used to determine luminescence of Rluc-*hPar2* donor, and filter 2 (515 ± 30nm) was used to measure *lrp6*-pEGFP-C1 (acceptor) luminescence, simultaneously. The data obtained are represented as the BRET^2^ ratio, with the BRET ratio for co-expressed Rluc-*hPar2* and *lrp6*-pEGFP-C1 constructs normalized against the BRET ratio found for the Rluc-*hPar2* expression construct alone in the same experiment [30].

BRET^2^ ratio =[(GFP acceptor emission at 515 ± 30nm)/(Rluc donor emission at 410 ± 70nm)] −cf.

The correction factor (cf), corresponds to (emission at 515 ± 30nm)/(emission at 410 ± 70nm), which is found when the Rluc-*hPar2* construct is expressed alone in the same experiment.

The BRET ratio was measured as mean ± standard deviation (SD) (mean ± SD), derived from three independent experiments using T Test statistical analysis. Data have been expressed as scattered and bar diagrams in excel drawing.

### Lef/Tcf luciferase reporter assay

RKO cells (0.5×10^5^) were plated in 12-well dishes and incubated overnight at 37°C. The cells were transfected with *hPar2-wt* or *hPar2* mutants (i.e. *hPar2-truncated, hPar2-356* or *hPar2-368*) plasmids, human Lef-1 TOPflash (Tcf Optimal Promoter + luciferase, T cell factor (Tcf) reporter plasmid containing two sets (the second set in reverse orientation) of three copies of the Tcf binding site upstream of the thymidine kinase (TK) minimal promoter and luciferase open reading frame using Fugene 6 transfection reagent (Boehringer-Mannheim). CMV/β-gal plasmid was cotransfected as an internal control for transfection efficiency. After 48 h transfection, the cells were washed in PBS and luciferase assay performed with the Luciferase Reporter System (Promega, Heidelberg, Germany) according to the manufacturer's instructions, and detected on a luminometer Mithras LB940, Berthold Technologies GmbH & Co. KG, Bad Wildbad, Germany).

### Immunofluorescence: cells

Either HCT-116 colon cancer or HEK-293 cells were plated on fibronectin (5μg/ml) coated coverslipsand transfected with *YFP-hPar2* and Lrp6. After overnight serum deprivation (0.2% BSA), the cells were activated by SLIGKV for various periods of time. Samples were then fixed with 4% paraformaldehyde in PBS containing 5% sucrose for 10 min and permeabilized with 0.5% triton X-100 in PBS for 3 min. Samples were fixed and stained with anti-LRP6 (H-300 rabbit polyclonal sc15399, Santa Cruz Biotechnology, Inc., Dallas Texas, USA) and anti GFP (for the detection of PAR_2_; SIGMA St Louis MO, USA). Cy2- and Cy3-conjugated anti-mouse IgG (Jackson Laboratories, West Grove, PA, USA) was used as asecond antibody. Cells were examined using laser confocal microscopy (Model 410, Zeiss, Oberkochen, Germany).

### Immunofluorescence: tumor tissue biopsy specimens

Paraffin embedded slides derived from PAR_2_ induced mammary gland tumors were deparaffinized and incubated in 3% H_2_O_2_. Antigen retrieval was carried out by heating (20 min) in a microwave oven in 10 mM Tris buffer containing 1 mM EDTA. After blocking the slides were incubated with the following primary antibodies: anti LRP6 (H-300 rabbit polyclonal sc15399, Santa Cruz Biotechnology, Inc. Dallas Texas, USA), anti-PAR_2_ (4μg/ml [SAM11] Santa Cruz), Cy2 conjugated anti-rabbit IgG, and Cy3-conjugated anti-mouse IgG antibodies (4μg/ml, Jackson Laboratories) were used as secondary antibodies. Nuclear staining was performed using DRAQ5 (4μM, Cell Signaling). Images were obtained using a Zeiss LSM 5 confocal microscope and analyzed with Zen software (Zeiss).

## SUPPLEMENTARY MATERIALS FIGURES AND TABLES


