# Supplementary material for: Low-density lipoprotein receptor-related protein 6 is a novel coreceptor of protease-activated receptor-2 in the dynamics of cancer-associated β-catenin stabilization
Source: Oncotarget. 2017 Mar 16;8(24):38650–67. doi: 10.18632/oncotarget.16246 (PMC5503561; doi:10.18632/oncotarget.16246)
Supplement: Supplementary file 1 [file oncotarget-08-38650-s001.pdf]

Low-density lipoprotein receptor-related protein 6 is a novel coreceptor of protease-activated receptor-2 in the dynamics of cancer-associated β-catenin stabilization

SUPPLEMENTARY MATERIALS

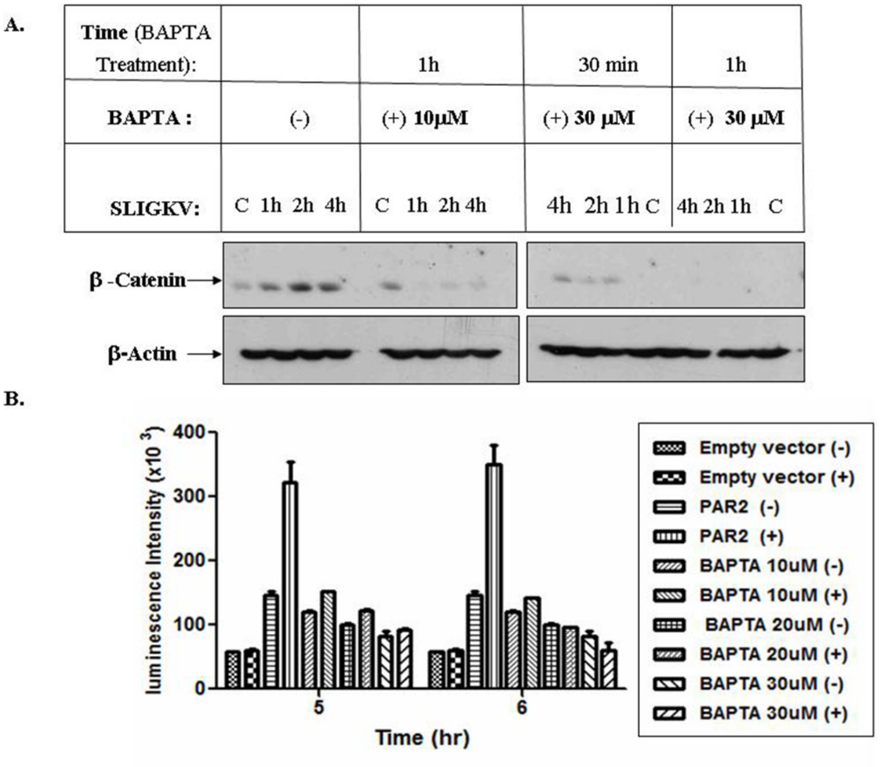

Supplementary Figure 1: BAPTA inhibits PAR_2-induced β-catenin stabilization levels and β-catenin transcriptional activity. **(A)** BAPTA inhibition of PAR_2 induced β-catenin levels. HU cells were transiently co-transfected with *hPar2* and *flg*-β-catenin plasmids. At various time points after SLIGKV activation, cell lysates were prepared and analyzed for levels of β-catenin. Pretreatment (30 min or 1 h) of the cells with BAPTA and treatment during the activation periods showed potent inhibition in β-catenin levels. **(B)** BAPTA inhibition of PAR_2-induced β-catenin transcriptional activity. TOPflash luciferase transcription activity was analyzed in HU cells following PAR_2 activation in the absence or presence of *hPar2 wt* following pretreatment of BAPTA (10-30 µM for 10 min prior to activation and following 5h or 6h SLIGKV activation). While following SLIGKV PAR_2 activation a marked increase in luciferase Lef/Tcf activity is observed ($p<0.01$), no increase was observed in the presence of BAPTA, where levels were similar to those of control non-activated PAR_2. Results were evaluated using GraphPad InStat software.

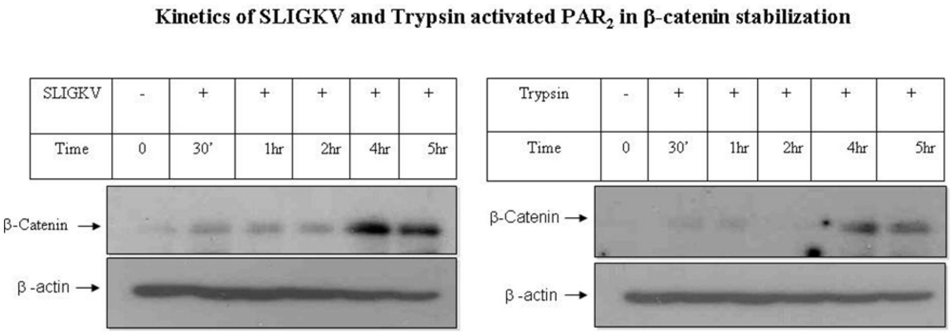

Supplementary Figure 2: Kinetics of SLIGKV and trypsin in PAR$_2$ induced β-catenin stabilization. HU cells were transiently co-transfected with *flg*-β-catenin and *hPar2* plasmids. The cells were activated by SLIGKV (100μM) and trypsin (50nM) for various time periods. Levels of β-catenin were determined by anti-*flg* antibodies. Protein levels were determined by anti β-actin levels.

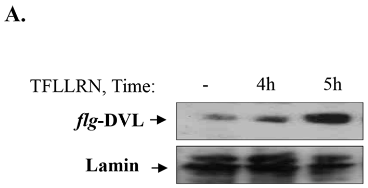

Immunofluorescence staining of DVL: Cellular localization following PAR_1 activation

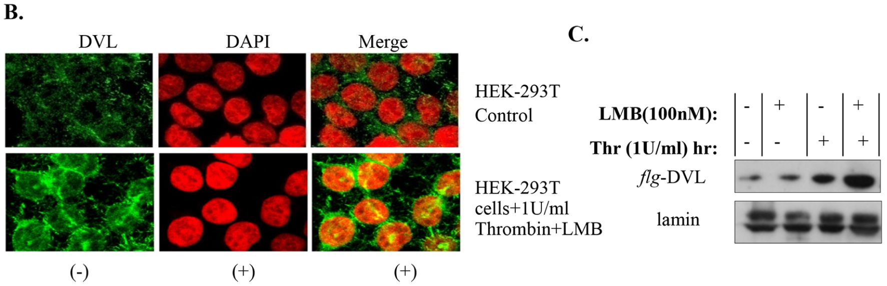

Supplementary Figure 3: DVL1 translocates to the nucleus following PAR_1 activation. **(A)** DVL1 accumulates in the nucleus following PAR_1 activation. HEK-293T cells were transiently transfected with *flg-dvl1*. The nuclear fraction was extracted and immunoblots were analyzed using either anti-*flg* and/or anti-lamin antibodies. DVL accumulated in the nucleus in a time dependent manner. **(B)** Immunoflorescence staining of DVL1. HEK-293T cells were transfected with *flg-dvl1* and activated with thrombin for 5 h following pretreatment with leptomycin B (LMB), a nuclear export inhibitor. Detection was carried out using anti-*flg* antibodies and fluorescent Cy3-conugated anti-mouse antibodies. Cell nuclei were counter-stained with DAPI. Following PAR_1 activation, nuclear localization of DVL1 was observed. **(C)** Levels of nuclear DVL1 following application of LMB and PAR_1 activation. Western blot analysis of nuclear DVL1 in the presence of LMB and following PAR_1 activation. While PAR_1 activation instigated potent DVL1 nuclear localization, in the presence of LMB, which blocks the nuclear export process, there was a marked increase in nuclear DVL following PAR_1 activation. This is shown using lamin as a marker for nuclear protein levels.

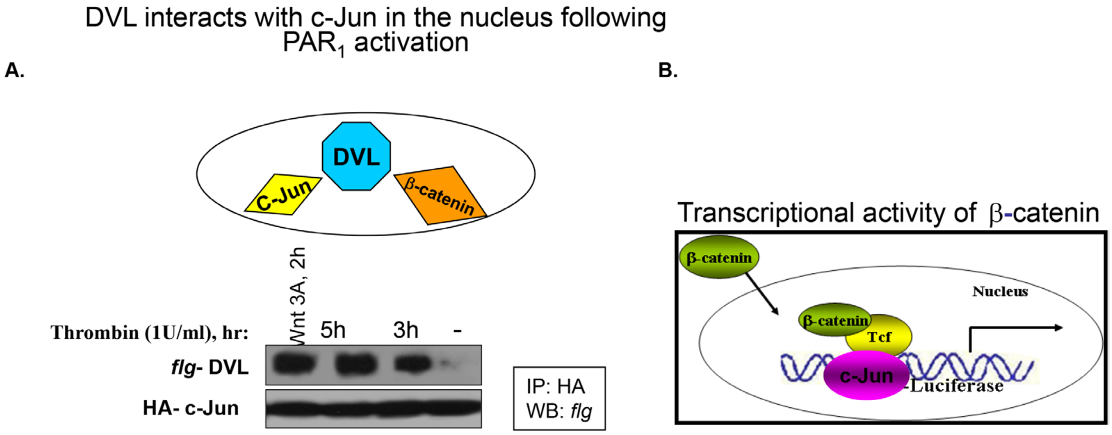

Supplementary Figure 4: DVL interacts with c-Jun in the cell nuclei following PAR$_1$ activation. (A) HEK-293T cell were transiently transfected with *flg*-dvl and HA-c-Jun plasmids. Following either thrombin or Wnt 3A activation, the nuclear fraction was isolated and subjected to immunoprecipitation analysis using anti-HA antibodies. DVL was detected following the application of anti-*flg* antibodies. **(B)** Scheme demonstrating interactions between β-catenin, DVL and c-Jun.

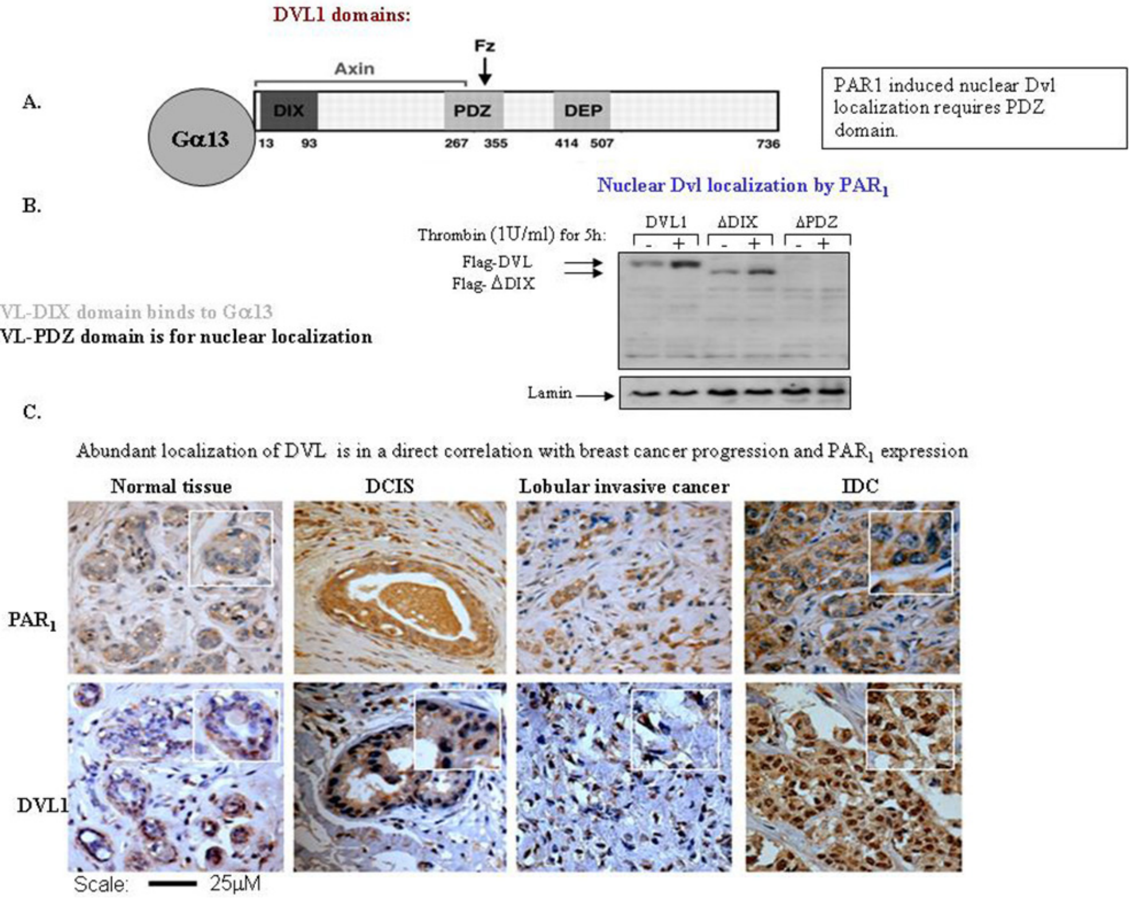

Supplementary Figure 5: PDZ domain is necessary for DVL nuclear localization. **(A)** Schematic presentation of DVL domains **(B)** HEK-293T cells were transiently transfected with ether *flg*- *wt* Dvl1, the deletion constructs (*flg*-ΔDIX-Dvl or *flg*-ΔPDZ-Dvl), or Dvl1 structural domain plasmids (*flg*-DIX, myc-PDZ, or myc-DEP). After PAR_1 activation, the nuclear fraction was prepared and immunoblots were analyzed using either anti-*flg*, anti-myc, or anti-lamin antibodies. The internalization of DVL to the nucleus was inhibited in the absence of the PDZ domain. The PDZ, but not the DIX domain, is necessary for PAR_1 induction of nuclear localization of DVL1. **(C)** Localization of PAR_1 and DVL1 in breast cancer tissue sections. Immunohistochemistry of paraffin-embedded breast tissue sections shows staining of both PAR_1 and DVL1 in the cytoplasm of breast cancer tissues, compared with very weak or no staining in normal-appearing tissue. In IDC, a very aggressive carcinoma, a massive straining of PAR_1 and DVL1 is observed in the cytoplasm as well as extensive expression of DVL in the nucleus. Data shown are representative of two independent experiments.

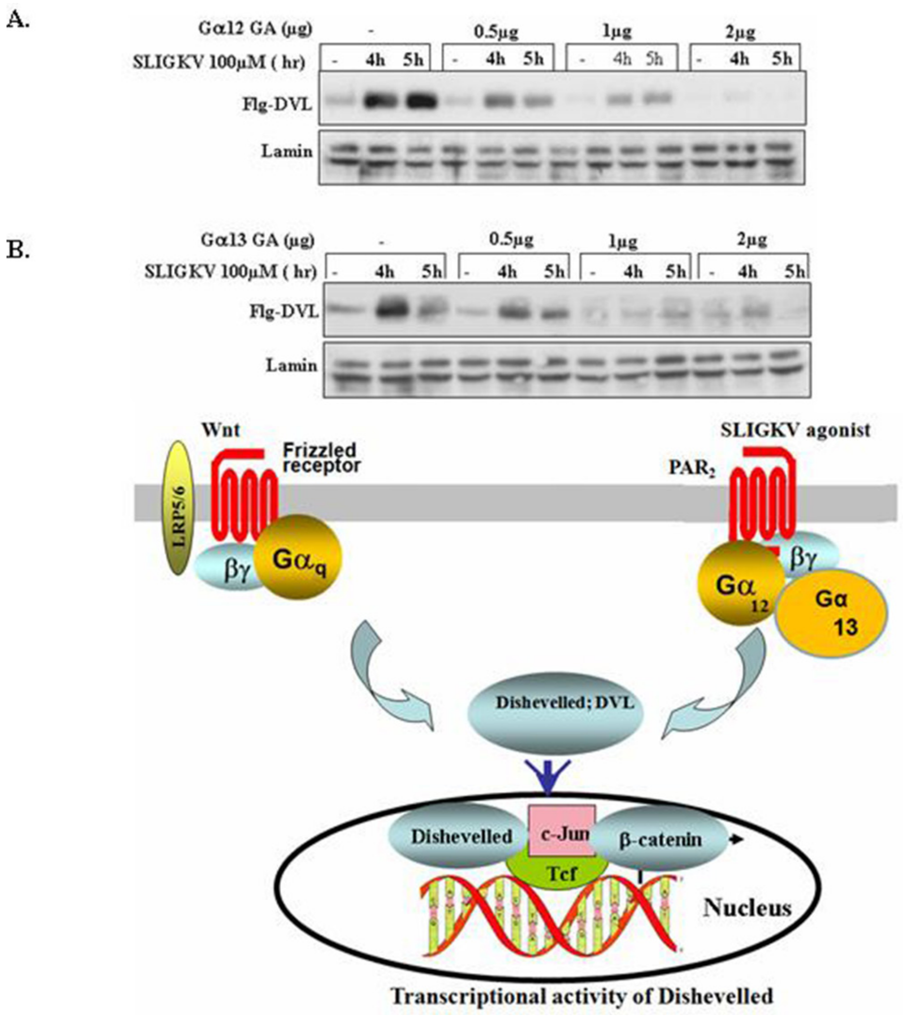

Supplementary Figure 6: Both Gα13 and Gα12 are involved in PAR_2-induced DVL nuclear localization. (A) A DNA form of $G\alpha_{12}$ inhibits DVL nuclear localization. HEK-293T cells were transiently transfected with *hPar2-wt, flg*-DVL, and $G\alpha_{12}$-GA dominant-negative (DN) plasmid. Following PAR_2 activation, immunoblots were analyzed using anti-*flg* (for *flg*-DVL), anti-lamin (or loading control) antibodies. $G\alpha_{12}$ GA potently inhibited PAR_2-induced DVL nuclear localization in a dose-dependent manner. **(B)** A DN form of $G\alpha_{13}$ inhibits DVL nuclear localization. HEK-293T cells were transiently transfected with *hPar2-wt, flg*-DVL, and $G\alpha_{13}$-GA DN plasmid. Following PAR_2 activation, immunoblots were analyzed using anti-*flg* (for *flg*-DVL) or anti-lamin (for loading control) antibodies. $G\alpha_{13}$-GA potently inhibited PAR_2-induced DVL nuclear localization in a dose-dependent manner.

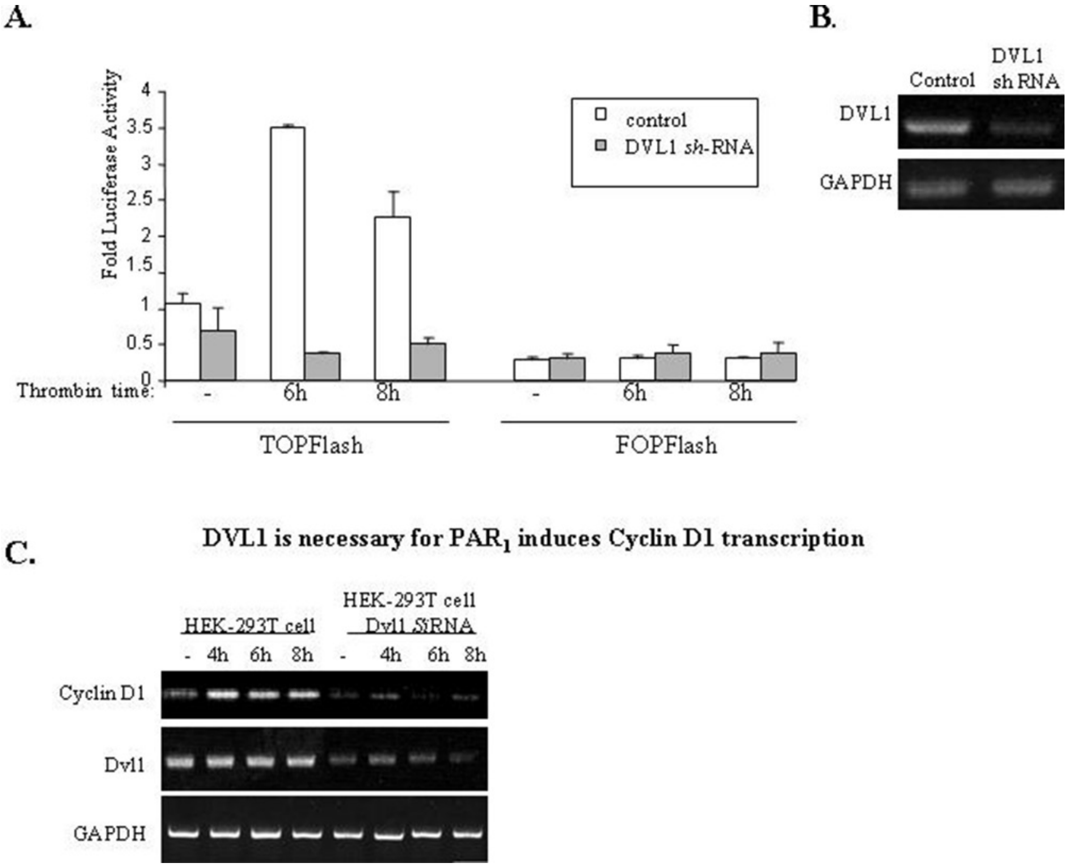

Supplementary Figure 7: DVL1 is a key mediator in PAR_1-induced transcriptional activity of Lef/Tcf. **(A)** DVL shRNA inhibits TOPFlash activity in HEK-293T cells. Naïve HEK-293T or HEK-293T cells infected with *sh dvl1* were seeded on 6-well plates and transfected with Lef1, TOPFlash, or FOPFlash as a negative control, and β-galactosidase plasmids. After 48 h the cells were treated with or without 1U/ml thrombin for 5 h. Results were normalized to the internal β-galactosidase activity to account for varying transfection efficiency. *sh*RNA of *dvl1* significantly decreased the transcriptional activity of Lef/Tcf mediated by PAR_1. **(B)** Dvl- *sh*RNA inhibits DVL levels. B. *dvl-sh*RNA effectively inhibits the RNA levels of *dvl1*. **(C)** DVL1 is necessary for PAR_1-induced Cyclin D1 transcription. Naïve HEK-293T cells and HEK-293T cells infected with *sh*RNA - *dvl1* were activated for 4, 6, or 8 hours by thrombin. RNA was isolated and RT-PCR was performed using primers for *cyclin d1, dvl1*, and GAPDH. Silencing knocked-down *dvl1* resulted in inhibition of cyclin d1 RNA levels.
